# Plasmid Copy Number Determination by Quantitative Polymerase Chain Reaction

**DOI:** 10.3797/scipharm.ISP.2015.02

**Published:** 2016-02-14

**Authors:** Anindyajati, A. Anita Artarini, Catur Riani, Debbie S. Retnoningrum

**Affiliations:** Laboratory of Pharmaceutical Biotechnology, School of Pharmacy, Institut Teknologi Bandung, Jalan Ganesha 10, 40132, Bandung, Indonesia

**Keywords:** Expression vector, Plasmid copy number, qPCR, Recombinant protein

## Abstract

Recombinant therapeutic proteins are biopharmaceutical products that develop rapidly for years. Recombinant protein production in certain hosts requires vector expression harboring the gene encoding the corresponding protein. *Escherichia coli* is the prokaryote organism mostly used in recombinant protein production, commonly using a plasmid as the expression vector. Recombinant protein production is affected by plasmid copy number harboring the encoded gene, hence the determination of plasmid copy number also plays an important role in establishing a recombinant protein production system. On the industrial scale, a low copy number of plasmids are more suitable due to their better stability. In the previous study we constructed pCAD, a plasmid derived from the low copy number pBR322 plasmid. This study was aimed to confirm pCAD’s copy number by quantitative polymerase chain reaction (qPCR). Plasmid copy number was determined by comparing the quantification signal from the plasmid to those from the chromosome. Copy number was then calculated by using a known copy number plasmid as a standard. Two pairs of primers, called *tdk* and *ori*, were designed for targeting a single gene *tdk* in the chromosome and a conserved domain in the plasmid’s *ori*, respectively. Primer quality was analyzed *in silico* using PrimerSelect DNASTAR and PraTo software prior to *in vitro* evaluation on primer specificity and efficiency as well as optimization of qPCR conditions. Plasmid copy number determination was conducted on *E. coli* lysates harboring each plasmid, with the number of cells ranging from 10^2^–10^5^ cells/μL. Cells were lysed by incubation at 95ºC for 10 minutes, followed by immediate freezing at −4°C. pBR322 plasmid with the copy number of ~19 copies/cell was used as the standard, while pJExpress414-*sod* plasmid possessing the high copy number pUC *ori* was also determined to test the method being used. *In silico* analysis based on primer-primer and primer-template interactions showed that both primer pairs were acceptable and were predicted to have good performance. Those predictions were in agreement with the *in vitro* test that gave a single band in the PCR product’s electropherogram and a single peak in DNA amplicon’s melting curve with a Tm value of 79.01 ± 0.11°C for the *tdk* primer and 81.53 ± 0.29°C for the *ori* primer. The efficiency of each primer was 1.95 and 1.97, respectively. The calculation result of pCAD’s copy number was 13.1 ± 0.3 copies/cell, showing that pCAD’s low copy number has been determined and confirmed. Meanwhile, it was 576.3 ± 91.9 copies/cell for pJExpress414-*sod*, in accordance with the hypothesis that pUC *ori* regulates the high copy number plasmid. In conclusion, the designed primers and qPCR conditions used in this study can be used to determine plasmid copy number for plasmids with pBR322 and pUC *ori*. The method should be tested further on plasmids harboring other type of *ori*.

## Introduction

Recombinant protein production could be increased by several strategies, one of which is by increasing copy number of gene encoding the corresponding protein. It could be done by choosing high copy number plasmid as the expression vector. However, the use of high copy number plasmid may lead to low stability, resulting in low cell productivity [1–4]. Regarding to those facts, low copy number plasmids are more suitable to be used as expression vector in industrial scale, due to its high stability [5].

In our previous study, we have successfully constructed pCAD, a pBR322-derived plasmid designed as vector expression for recombinant protein production in *Escherichia coli* (unpublished data). Since it was derived from pBR322, it is expected to have low copy number plasmid of around 15–20 copies/cell, similar to pBR322 itself [6]. Despite its high protein expression, the low copy number of the plasmid has not been confirmed yet. Since the determination of plasmid copy number plays an important role in establishing recombinant protein production system, a method to determine plasmid copy number has to be developed.

There are several techniques available for plasmid copy number determination, such as agarose gel electrophoresis and quantitative polymerase chain reaction (qPCR) [6, 7]. Between those two, qPCR is relatively more sensitive and less time-consuming. Existing qPCR method for plasmid copy number determination requires the use of *bla* gene in the plasmid, causing limitation of the method, applicable to only plasmids having *bla* gene [6, 8, 9]. Here we developed a plasmid copy number determination method by qPCR using plasmid replication machinery region as the target for plasmid amplification. The method is expected to be able to be used to confirm pCAD’s ow copy number, and also applicable to a broad range of plasmids.

## Results and Discussion

pCAD being determined in this study is a plasmid being constructed as expression vector in recombinant protein production using *E. coli* as the host. Since the plasmid is derived from pBR322, it is expected to have low copy number also, ranged from 15–20 [6]. To confirm the low copy number of the plasmid, plasmid copy number determination was conducted using qPCR. The determination was based on the signal of plasmid amplification product compared to the signal of chromosome amplification product, representing the number of plasmid copy per cell. The ratio of plasmid to chromosome (P/C) was then used as the value representing plasmid copy number, using known-copy-number plasmid as a standard. DNA amplification in qPCR requires a set of primers that are specific towards target and also have good primer-primer interaction, resulting in a good primer efficiency. Besides, length of the product was also limited in order to avoid too long elongation step, leading to the decrease of amplification eficiency [10].

Two set of primers were designed. The first set, P*tdk*, was targeted to amplify a part of *tdk*, a single copy gene encoding thymidine kinase in *E. coli* chromosome. While the second one, P*ori*, was targeted to amplify a conserved region of replication machinery (*ori* along with its regulator) in pBR322 and pUC *ori*. The primer was targeted to conserved region of pBR322 and pJExpress414-*sod* replication machinery. The targeted region was expected to be universal, allowing the method to be applied to a board range of plasmids. It does not need the existence of certain gene, such as ampicillin resistance gene (*bla*) that was commonly used as target in plasmid copy number determination using qPCR [6, 8, 9]. Two set of primers designed were then evaluated further.

Primer quality evaluation was done *in silico* using PrimerSelect DNASTAR to analyze primer-template interaction and PRaTo software to analyze primer quality solely along with primer-primer interaction. PrimerSelect DNASTAR analysis showed that both primer set were specific towards each target (data not shown). Meanwhile, PRaTo analysis showed that both primer set were promising for qPCR (Tab. 1).

**Tab. 1:**
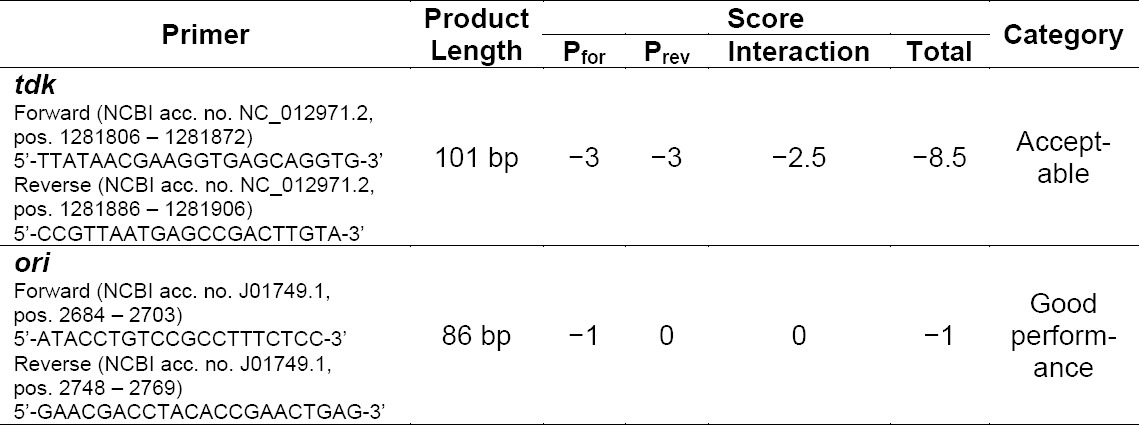
Evaluation of Primer Quality using PRaTo Software

**Fig. 1:**
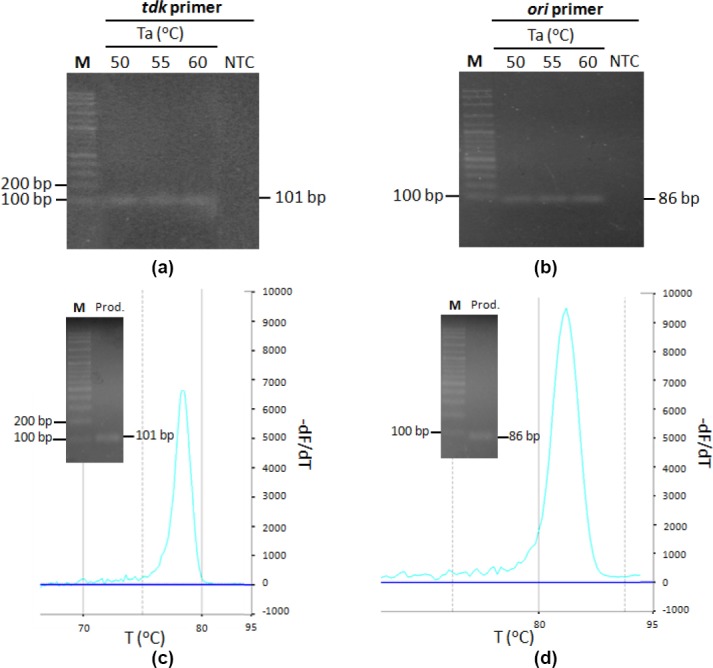
P*tdk* and P*ori* specificity test. PCR product electrophoretogram of (a) P*tdk* to *E. coli* BL21(DE3) chromosome, (b) P*ori* to pCAD plasmid PCR product; melting curve analysis and electrophoretogram of (c) P*tdk*, (d) P*ori* qPCR product. M marker, NTC non-template control, Prod. qPCR product.

qPCR condition optimization was conducted to determine the most optimum Ta. PCR with various Ta was run, and results showed that PCR reaction performed well in all tested condition (Fig. 1). Hence, Ta of 60ºC was then used to avoid unspecific binding and primer secondary structure formation tend to occur in lower temperature. To confirm primer specificity that had been analyzed *in silico* previously, conventional PCR was performed as preliminary test, followed by qPCR product melting curve analysis. Both results showed that *tdk* and *ori* primer set were specific towards each of its target, yielding single product (Fig. 1). Although the specificity of both primer set towards each target template has been confirmed, cross-annealing of primer should also be tested, due to the sample for copy number determination being cell lysate, that is a mixture between chromosome and plasmid. The occurence of cross reaction may result in unwanted increase of signal, resulting in miscalculation of P/C ratio.

**Fig. 2:**
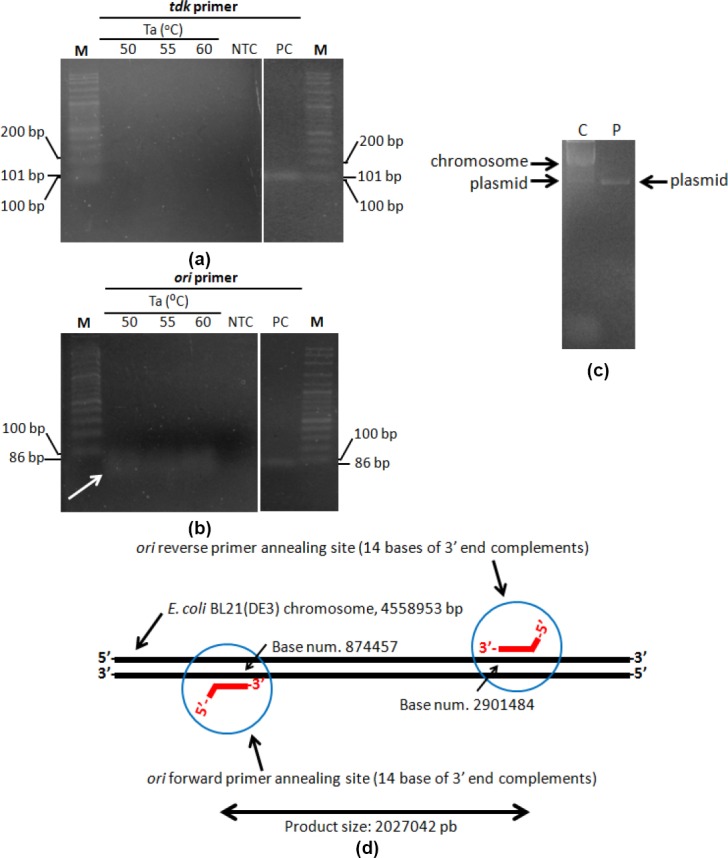
P*tdk* and P*ori* cross-annealing test. PCR product electrophoretogram of (a) P*tdk* to plasmid isolate, (b) P*ori* to chromosome (genomic DNA) isolate of recombinant *E. coli* BL21(DE3) harboring plasmid; (c) Chromosome (genomic DNA) and plasmid isolate of recombinant *E. coli* BL21(DE3) harboring plasmid; (d) position of P*ori* annealing site to *E. coli* BL(21)DE3 chromosome according to BLAST NCBI. M marker, NTC non-template control, PC positive control, C chromosome (genomic DNA) isolate of recombinant *E. coli* BL21(DE3) harboring pCAD, P plasmid isolate.

Primer cross-annealing was tested by reacting each primer set to the template that is not their target. PCR was run for amplifying isolated chromosome with P*ori* and isolated plasmid with P*tdk*. The electrophoretogram showed that there was no product band in PCR of isolated plasmid amplification using P*tdk*. Product band appeared in electrophoretogram of isolated chromosome amplification using P*ori* was caused by traces of plasmid being isolated in genomic DNA isolation process, as shown in the electrophoretogram of genomic DNA isolation product (Fig. 2). It was also supported by *in silico* analysis using BLAST NCBI showing that 3’-ends of P*ori* could not possibly anneal to *E. coli* chromosome to produce amplification giving a 86-bp product as shown in the electrophoretogram (Fig. 2).

**Fig. 3:**
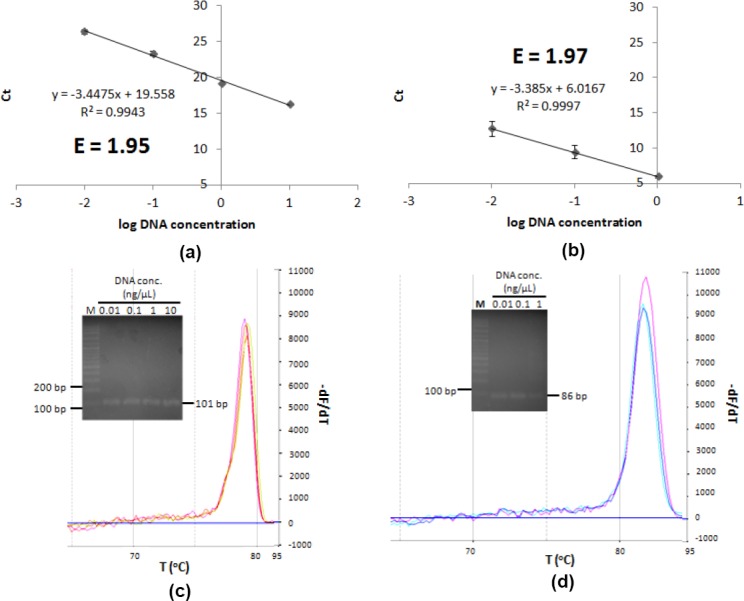
P*tdk* and P*ori* efficiency determination. Graph of Ct correlation to log DNA concentration of (a) P*tdk*, (b) P*ori*; melting curve analysis and electrophoretogram of (c) P*tdk*, (d) P*ori* qPCR product. M marker.

Following the confirmed primers specificity, primer efficiency was determined. Primer efficiency is a value representing primer performance in DNA amplification reaction. Primer efficiency of 2.00 represents 100% amplification efficiency, meaning that the reaction successfully amplified a template into two products in a cycle. So, it was expected that the efficiency of primers being used will be close to 2.00, ranged from 1.85 to 2.05 [10]. Too high primer efficiency indicates either pippetting error or the existence of PCR inhibitor being diluted in the sample gradual dilution, affecting the gradient of the linear equation plotted. Too low primer efficiency indicates poor primer performance may caused by several factors, such as unspecific primer and too long PCR product [11]. The results show that P*tdk* and P*ori* have good efficiency, that are 1.95 and 1.97, respectively (Fig. 3). Product melting curve analysis and electrophoretogram showed that amplification gave single product up to template concentration of 10 ng/μl for chromosome and 1 ng/μl for plasmid with Tm value of 79.01 ± 0.11°C and 81.53 ± 0.29°C, respectively (Fig. 3).

**Fig. 4:**
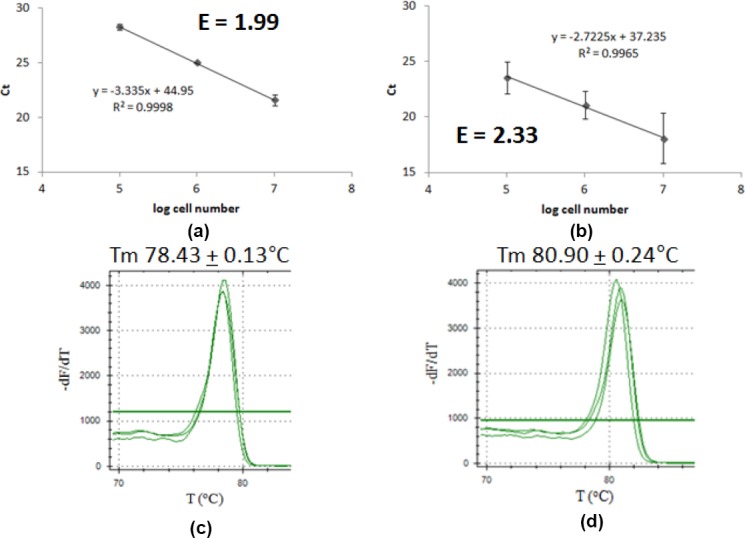
qPCR result for pBR322 P/C ratio determination. Graph of Ct correlation to cell number of (a) chromosome as target, (b) pBR322 plasmid as target; melting curve analysis of (c) chromosome as target, (d) pBR322 plasmid as target.

Since the specificity and efficiency of primers had been confirmed, qPCR for plasmid copy number determination was then conducted. Defined number of cell lysate was kept frozen prior to thawing right before mixing of qPCR components, expected to give the lowest number of degraded DNA [12]. Data analysis and calculation gave pBR322 P/C ratio of 1.94 ± 0.16 (Tab. 2). The value was used as standard representing pBR322 known copy number of ~19 copies/cell (Tab. 2). Product melting curve analysis showed that the amplification reaction of P*tdk* and P*ori* each gave single product with Tm value of 78.43 ± 0.13°C and 80.90 ± 0.24°C, respectively (Fig. 4).

**Tab. 2:**
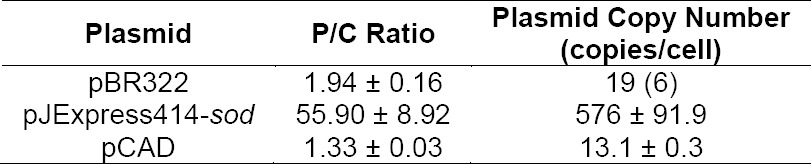
Plasmid Copy Number Determination by qPCR

**Fig. 5:**
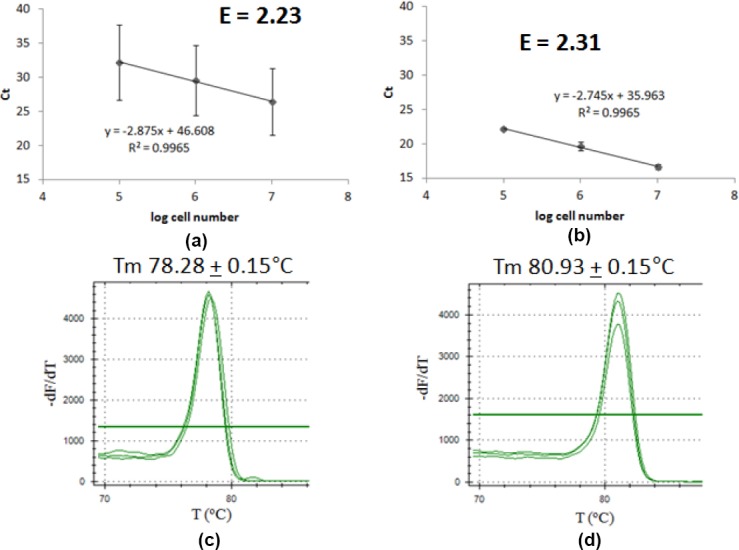
qPCR result for pJExpress414-*sod* plasmid copy number determination. Graph of Ct correlation to cell number of (a) chromosome as target, (b) pJExpress414-*sod* plasmid as target; melting curve analysis of (c) chromosome as target, (d) pJExpress414-*sod* plasmid as target.

In order to test the method, plasmid copy number of pJExpress414-*sod* possessing pUC *ori* was determined. pUC *ori* supposed to have high copy number of ~500–700 copies/cell [13]. Determination using qPCR method in this study gave pJExpress414-*sod* P/C ratio of 55.90 ± 8.92, representing plasmid copy number of 576 ± 91.9 copies/cell, matched its theoretical value (Tab. 2). Product’s melting curve showed that both amplification reactions gave single product with Tm value of 78.28 ± 0.15°C for chromosome and 80.93 ± 0.15°C for plasmid (Fig. 5). Hence, the method had been proven to be able to be used to determine plasmid with high copy number.

**Fig. 6:**
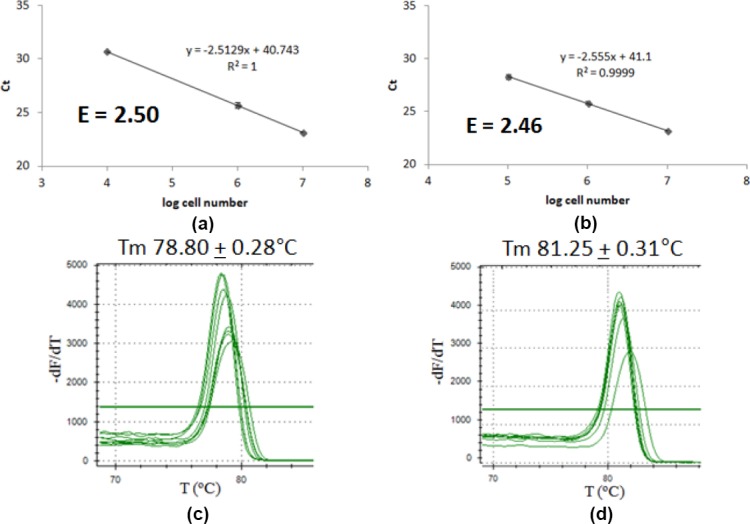
qPCR result for pCAD plasmid copy number determination. Graph of Ct correlation to cell number of (a) chromosome as target, (b) pCAD plasmid as target; melting curve analysis of (c) chromosome as target, (d) pCAD plasmid as target.

Plasmid copy number of pCAD was then determined. Product melting curve analysis showed that the amplification reaction of P*tdk* and P*ori* each gave single product with Tm value of 78.80 ± 0.28°C and 81.25 ± 0.31°C, respectively (Fig. 6). Data analysis and calculation gave pCAD P/C ratio of 1.33 ± 0.03 (Fig. 6, Tab. 2). Using the standard, this value was converted to plasmid copy number, giving the value of 13.1 ± 0.3 copies/cell (Tab. 2). According to the hypothesis, pCAD that is derived from pBR322 should have low copy number, around 15–20 copies/cell. The lower value of pCAD copy number could possibly because of the existence of inserted gene regulated by autoinduction promoter that does not exist in pBR322. Overexpression of recombinant protein may lead to the decrease of plasmid copy number in a cell, let alone if it is toxic [13].

In plasmid copy number determination of all plasmids, almost all the qPCR efficiency value was higher than 2.05. It could possibly be caused by the existence of PCR inhibitors in the mixture, regarding to the fact that the sample being used was cell lysate processed without DNA extraction. One of the possible substance acting as PCR inhibitor was lipopolysaccharide as component of *E. coli* cell wall [14]. Gradual dilution of cell number in suspension consequently diluted the PCR inhibitor, affecting the gradient of correlation of signal produced to number of templates that may lead to an increase in efficiency [11]. Lower concentration of template, being number of cells in this case, could be done to further optimize this method, in order to omit the effect of PCR inhibitors.

From the results obtained, we conclude that the designed primers and qPCR condition can be used to determine plasmid copy number for plasmids with pBR322 and pUC *ori*. By this method, we revealed that pJExpress414-*sod* has the copy number of ~576 ± 92 copies/cell, while pCAD has the copy number of 13 ± 0.3 copies/cell. For further study, optimization of this method by using lower concentration of template can be used to minimize PCR inhibitors effect. The method should also be tested on plasmids *ori* types other than pBR322 and pUC.

## Experimental

### Strains

Recombinant *E. coli* BL21(DE3) harboring pBR322 was used as standard, while recombinant *E. coli* BL21(DE3) harboring pJExpress414-*sod* was used to test the method. The plasmid copy number pCAD in recombinant *E. coli* TOP10 was then determined. All bacteria harboring plasmids were cultured in LB medium at 37°C with addition of 100 μg/mL of ampicillin (Bernofarm).

### Primer Sets Design for qPCR

Two sets of primers were designed, each targeting a single copy DNA sequence in either plasmid or chromosome. Primer design was conducted using PrimerSelect DNASTAR software. The first set of primer, donated as P*tdk*, is specific to the thymidine kinase, a single copy gene in *E. coli* BL21(DE3) chromosome (NCBI, Access No. CP001509.3). The second set of primer, that is P*ori*, is specific to a part of conserved domain of plasmid replication machinery in pBR322 and pUC-derived plasmids (NCBI, Access No. J01749.1; DNA 2.0, pJexpress414:30318 - SoeEq_V4).

### qPCR Condition Optimization and Primer Sets Quality Testing

#### In silico Testing

Primer sets quality was first tested *in silico* using PrimerSelect DNASTAR and PRaTo softwares prior to *in vitro* testing. PrimerSelect DNASTAR was used to evaluate primer-template interaction, while PRaTo was used to evaluate primer quality alone and primer-primer interaction. PrimerSelect DNASTAR could analyze the possible anneling sites of primer set on the template. Specific primer set should only anneal on a single site. PRaTo analysis results were interpreted based on the score obtained, being categorized as either best-performing primer set (0 to −6), acceptable primer set (−7 to −11), and should-be-discarded-primer set (less than −11) [15].

#### Primer Specificity

Each primer set was run for PCR using chromosome as template for P*tdk* and plasmid as template for P*ori*. Chromosome was isolated using Wizard^®^ Genomic DNA Purification Kit (Promega) and plasmid was isolated using High-Speed Plasmid Mini Kit (Geneaid). PCR was run in varying annealing temperatures (Ta), that were 50, 55, and 60ºC. Highest Ta yielding a single product analyzed using agarose gel electrophoresis was then further used in qPCR. Primer specificity was evaluated based on qPCR product melting curve analysis also. Specific primer set would give single sharp peak in the melting curve analysis of qPCR product and a single band in 2% agarose gel electrophoresis of both PCR and qPCR products. Cross annealing (P*tdk* targeting plasmid and P*ori* targeting chromosome) was also tested.

#### Primer Efficiency

For the determination of primer efficiency, qPCR was run using various template concentration, ranging from 0.01 to 10 ng/μl. Data acquired were analyzed using PikoReal2 software. Primer efficiency was calculated by plotting the correlation of cycle threshold (Ct) to log DNA template concentration, giving the equation y = Bx + A. Primer efficiency was obtained using the equation E = 10^(−1/B)^. Good primer efficiency ranged between 1.85 to 2.05 [10].

### Sample Preparation

Samples used in plasmid copy number determination were recombinant bacterial cell lysates. Overnight cell culture was centrifuged. The pellet was then resuspended in sterile PBS 1x pH 7.4 to give a cell suspension having OD_600_ of ~1, followed by a series of dilution of up to 10^-9^ times. As much as 100 μl each of 10^-9^, 10^-8^, 10^-7^, 10^-6^, and 10^-5^ diluted suspension culture was spread on solid medium prior to overnight incubation for viable cell count. Simulatenously, cells were lysed by 95ºC incubation for 10 minutes, followed by immediate freezing in −20°C [12]. As much as 3 μl suspension of lysed cells with the concentration ranged from 10^2^–10^5^ cells/μl was then amplified using KAPA SYBR^®^ FAST qPCR Master Mix (2x) Universal and both set of primers in separated reaction. Data obtained were analyzed using CFX Manager 2.0 software.

### Data Analysis

The correlation of Ct to log cell number was plotted to determine the ratio of plasmid to chromosome per cell (P/C) based on the equation below [12]. Ec = P*tdk* efficiency targeting chromosome, Ep = P*ori* efficiency targeting plasmid, Ct_c_ = Ct of P*tdk* targeting chromosome, Ct_p_ = Ct of P*ori* targeting plasmid.


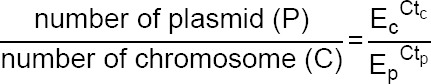


pBR322 plasmid was used as standard, hence the value of P/C was assumed to represent plasmid copy number of 19 copies/cell [6]. pJExpress414-*sod* having pUC *ori* was analyzed to test the method. Plasmids having pUC *ori* have high copy number, ranged from ~500 to 700 copies/cell [13]. pCAD copy number was then determined.
